# Biallelic *NDUFA9* variants cause a progressive neurodevelopmental disorder with prominent dystonia and mitochondrial complex I deficiency

**DOI:** 10.1093/braincomms/fcaf369

**Published:** 2025-09-23

**Authors:** Francesca Magrinelli, Lucie S Taylor, Sahar Sedighzadeh, Dalila Moualek, Mariasavina Severino, Daniel N Grba, Charlotte L Alston, Michael Champion, Ali Reza Tavasoli, Karine Lascelles, Vykuntaraju K Gowda, Varunvenkat M Srinivasan, Sahand Tehrani Fateh, Mohammad Kordi-Tamandani, Ali Khajeh, Saeedeh Yaghoubi, Natalia Dominik, Meisam Babaei, Mohsen Javadzadeh, Jamileh Rezazadeh Varaghchi, Mohammad Miryounesi, Ehsan Ghayoor Karimiani, Meriem Tazir, Lamia Ali Pacha, Kailash P Bhatia, Robert W Taylor, Henry Houlden, Reza Maroofian

**Affiliations:** Department of Clinical and Movement Neurosciences, UCL Queen Square Institute of Neurology, University College London, London WC1N 3BG, United Kingdom; Mitochondrial Research Group, Translational and Clinical Research Institute, Faculty of Medical Sciences, Newcastle University, Newcastle upon Tyne NE2 4HH, United Kingdom; NHS Highly Specialised Service for Rare Mitochondrial Disorders, Newcastle upon Tyne Hospitals NHS Foundation Trust, Newcastle upon Tyne NE1 4LP, United Kingdom; Department of Biological Science, Faculty of Science, Shahid Chamran University of Ahvaz, Ahvaz 6135783151, Iran; Dr Shahrooei Laboratory, Tehran 1476693993, Iran; Service de Neurologie, CHU Mustapha Bacha, Alger 16000, Algeria; Université Benyoucef Benkhedda, Alger 16000, Algeria; Neuroradiology Unit, IRCCS Istituto Giannina Gaslini, Genova 16147, Italy; Medical Research Council Mitochondrial Biology Unit, University of Cambridge, Cambridge CB2 0XY, United Kingdom; Mitochondrial Research Group, Translational and Clinical Research Institute, Faculty of Medical Sciences, Newcastle University, Newcastle upon Tyne NE2 4HH, United Kingdom; NHS Highly Specialised Service for Rare Mitochondrial Disorders, Newcastle upon Tyne Hospitals NHS Foundation Trust, Newcastle upon Tyne NE1 4LP, United Kingdom; Department of Paediatric Inherited Metabolic Disease, Evelina London Children's Hospital, Guy's and St Thomas’ NHS Foundation Trust, London SE1 7EH, United Kingdom; Department of Neurology, Barrow Neurological Institute at Phoenix Children’s Hospital, Phoenix, AZ 85016, USA; Myelin Disorders Clinic, Pediatric Neurology Division, Children's Medical Center, Tehran University of Medical Sciences, Tehran 1419733151, Iran; Children's Neuroscience Centre, Evelina London Children's Hospital, Guy's and St Thomas’ NHS Trust, London SE1 7EH, United Kingdom; Department of Pediatric Neurology, Indira Gandhi Institute of Child Health, Near NIMHANS, Bengaluru, Karnataka 560029, India; Department of Pediatric Neurology, Indira Gandhi Institute of Child Health, Near NIMHANS, Bengaluru, Karnataka 560029, India; School of Medicine, Tehran University of Medical Sciences (TUMS), Tehran 1416634793, Iran; Department of Biology, Faculty of Science, University of Sistan and Baluchestan, Zahedan 9816745785, Iran; Children and Adolescent Health Research Center, School of Medicine, Resistant Tuberculosis Institute, Zahedan University of Medical Sciences, Zahedan 9816743463, Iran; Department of Pediatrics, School of Medicine, Children and Adolescents Health Research Center, Research Institute of Cellular and Molecular Science in Infectious Diseases, Zahedan University of Medical Sciences, Zahedan 9816743463, Iran; Department of Neuromuscular Diseases, UCL Queen Square Institute of Neurology, London WC1N 3BG, United Kingdom; Department of Pediatrics, North Khorasan University of Medical Science, Bojnourd 9417694735, Iran; Department of Pediatric Neurology, Shahid Beheshti University of Medical Sciences, Tehran 1983969367, Iran; Hasti Genetic Counseling Center of Welfare Organization of Southern Khorasan, Birjand 9719866879, Iran; Center for Comprehensive Genetic Services, Shahid Beheshti University of Medical Sciences, Tehran 1985717413, Iran; Department of Medical Genetics, Faculty of Medicine, Shahid Beheshti University of Medical Sciences, Tehran 1985717443, Iran; Department of Neuromuscular Diseases, UCL Queen Square Institute of Neurology, London WC1N 3BG, United Kingdom; Service de Neurologie, CHU Mustapha Bacha, Alger 16000, Algeria; Université Benyoucef Benkhedda, Alger 16000, Algeria; Service de Neurologie, CHU Mustapha Bacha, Alger 16000, Algeria; Université Benyoucef Benkhedda, Alger 16000, Algeria; Department of Clinical and Movement Neurosciences, UCL Queen Square Institute of Neurology, University College London, London WC1N 3BG, United Kingdom; Mitochondrial Research Group, Translational and Clinical Research Institute, Faculty of Medical Sciences, Newcastle University, Newcastle upon Tyne NE2 4HH, United Kingdom; NHS Highly Specialised Service for Rare Mitochondrial Disorders, Newcastle upon Tyne Hospitals NHS Foundation Trust, Newcastle upon Tyne NE1 4LP, United Kingdom; Department of Neuromuscular Diseases, UCL Queen Square Institute of Neurology, London WC1N 3BG, United Kingdom; Department of Neuromuscular Diseases, UCL Queen Square Institute of Neurology, London WC1N 3BG, United Kingdom

**Keywords:** lactic acidosis, Leigh syndrome, mutational hotspot, seizures, spasticity

## Abstract

Biallelic *NDUFA9* variants have hitherto been associated with disease in four individuals. Hence, clinicogenetic features of *NDUFA9*-related disorder remain largely unexplored. To delineate the pheno-genotypic spectrum of *NDUFA9*-related disorder, we screened genetic databases worldwide and collected phenotypic data on individuals with biallelic *NDUFA9* variants, which were functionally investigated when possible. Eight new and four reported cases were identified. Neurodevelopmental delay followed by motor deterioration and seizures were the most common presenting features. Neurodevelopmental disorder was observed in 90% of cases surviving beyond the age of 4 months. Neurological deterioration always started in the first decade. Among ten affected surviving beyond early infancy, major clinical features included dystonia (100%), feeding difficulties/dysphagia/failure to thrive and pyramidal signs (80%), seizures and muscle weakness/atrophy (70%), and moderate-to-severe intellectual disability (60%). All showed basal ganglia MRI signal alterations, with atrophy (50%) and swelling (25%). Four individuals died by the age of 13 years. In addition to four known variants, we identified five new *NDUFA9* variants and pinpointed Arg360 (NP_004993.1) as a mutational hotspot. Protein modelling suggested that variants cause NADH:ubiquinone oxidoreductase subunit A9 (NDUFA9) misfolding and/or disruption of binding interfaces. Loss of fully assembled complex I with decreased steady-state NDUFA9 levels and/or complex I activity was documented in fibroblasts from three affected individuals. Our study strengthens the evidence that biallelic *NDUFA9* variants cause mitochondrial complex I deficiency presenting with a broad spectrum of progressive neurodevelopmental disorder, often accompanied by prominent dystonia, and a characteristic Leigh syndrome MRI pattern.

## Teaser

Magrinelli *et al*. delineated the pheno-genotypic spectrum of *NDUFA9*-related mitochondrial disease based on eight new and four previously reported cases. Biallelic *NDUFA9* variants cause mitochondrial complex I deficiency, with clinical presentations ranging from a progressive neurodevelopmental disorder with prominent dystonia to isolated dystonia accompanied by basal ganglia MRI signal abnormalities.

## Graphical Abstract

**Graphical Abstract:**
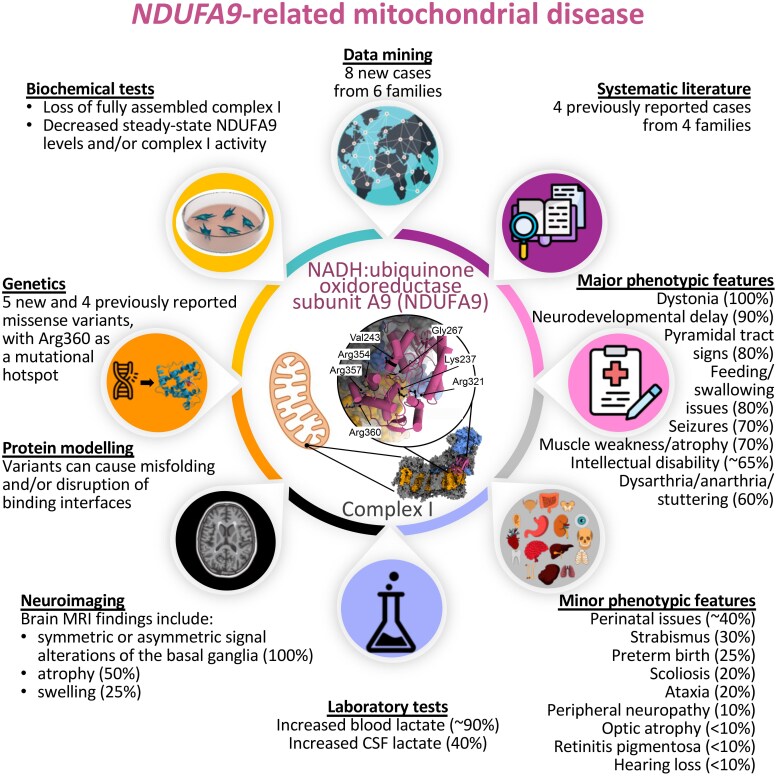

## Introduction

Complex I is the major multiprotein enzyme of the mitochondrial respiratory chain.^1^ Besides representing the main entry of electrons to the oxidative phosphorylation system (OXPHOS), it couples quinone reduction by NADH (reduced nicotinamide-adenine dinucleotide) to proton translocation across the inner mitochondrial membrane.^2,3^ The resulting electrochemical gradient ultimately drives protons back through the membrane via ATP synthase, which phosphorylates adenosine diphosphate (ADP) to ATP.^1^ Complex I consists of 14 catalytic core and 30 accessory subunits, 37 of which coded by nuclear DNA.^1,4,5^ Subunits are assembled in three functional modules: Q-module (ubiquinone reduction), N-module (NADH dehydrogenase) and P-module (proton translocation).^3^

Encoded by the homonymous nuclear gene, NADH:ubiquinone oxidoreductase subunit A9 (NDUFA9) is a 39-kDa Q-module supernumerary subunit which is conserved in eukaryotes.^6^ NDUFA9 is synthetized in the cytosol and imported into mitochondria by an N-terminal presequence which is cleaved by matrix proteases.^6^  *In vitro*, NDUFA9 is suggested to anchor complex I membrane and matrix arms by contributing to stability of a later stage assembly intermediate, as documented by gene knockout in human embryonic kidney 293T (HEK293T) cells.^1,6^ Additionally, NDUFA9 is crucial for complex I function, as proven by the inability of the same cells to grow in galactose-containing medium.^6^ Given its location and membrane remodelling capacity, NDUFA9 has been implicated in recruiting the membrane-bound substrate ubiquinone-10 in complex I function.^7,8^ Finally, NDUFA9 has been implicated in regulating complex I in ischemia–reperfusion injury.^9^

Leigh syndrome (LS) spectrum represents the most frequent presenting phenotype of childhood-onset mitochondrial disease, being causally linked to variants in over 110 genes across nuclear and mitochondrial genomes.^10^ Biochemical defects in complex I cumulatively account for most cases of early-onset mitochondrial disease in humans.^11-13^ Nevertheless, biallelic *NDUFA9* variants have hitherto been linked to disease phenotypes in only four individuals, namely two cases of lethal infantile LS^14-16^ and two cases of childhood-onset generalized dystonia.^15,17^

We herein report eight new cases of *NDUFA9*-related mitochondrial disease from six unrelated families, harbouring five novel missense *NDUFA9* variants, three of which were investigated with functional studies of patient-derived fibroblasts.

## Material and methods

### Proband identification, phenotypic characterization and sampling

Individuals with homozygous or compound heterozygous *NDUFA9* variants were identified by interrogating the University College London (UCL) Queen Square exome repository and next-generation sequencing datasets curated from national and international collaborators. Exome sequencing was performed on DNA extracted from whole blood across six different research and diagnostic laboratories, each employing slightly varying methodologies for sequencing and data analysis. We used Sanger sequencing to confirm *NDUFA9* variants identified by next-generation sequencing in the probands and to perform segregation analysis on available genomic DNA from their family members, including both parents of seven affected individuals in Families 1–2–3–4–5 (12 individuals), two probands affected siblings (P2 in Families 1 and 2), and eight probands unaffected siblings in Families 1–2–3. Detailed clinical data were collected using a standardized proforma completed by referring clinicians for all affected individuals. Video recordings of neurological examinations were reviewed by movement disorder specialists (FM, KPB) when available. Neuroimaging was assessed by an experienced paediatric neuroradiologist (MS).

**Figure 1:**
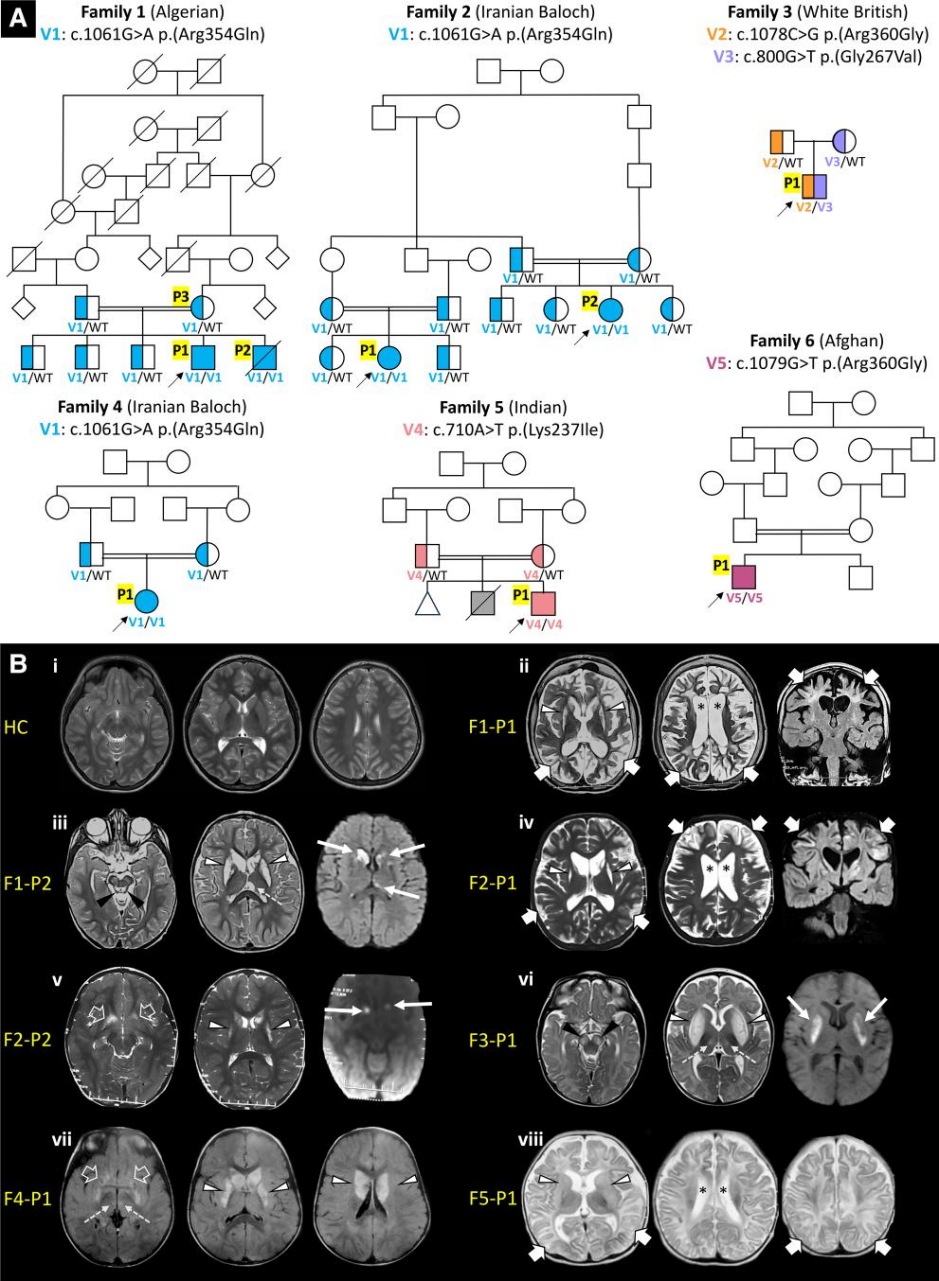
**Pedigrees and neuroimaging findings of eight new individuals with *NDUFA9*-related mitochondrial disease.** (**A**) Pedigrees of eight new cases of *NDUFA9*-related mitochondrial disease reported in this study with their ethnicity and results of segregation analysis of *NDUFA9* variants. Probands are identified by arrows. Fully filled coloured symbols indicate affected individuals (P#) carrying *NDUFA9* variants in the homozygous or compound heterozygous state. Half-filled coloured symbols identify asymptomatic heterozygous carriers of a mutant *NDUFA9* allele. Each colour represents a different *NDUFA9* variant (V#; see also Fig. 2 and Supplementary File 3). A fully filled grey symbol represents lack of molecular diagnosis in a family member with congenital heart disease. Deceased individuals are identified by a diagonal line. (**B**) Brain MRI examinations of a healthy control (HC) (**i**) and of affected individuals F1-P1 at 7 years of age (**ii**), F1-P2 at 4 years (**iii**), F2-P1 at 5 years of age (**iv**), F2-P2 at 3 years of age (**v**), **F**3-P1 at 6 months of age (**vi**), F4-P1 at 2 years of age (**vii**), and F5-P1 at 5 months of age (**viii**). There are bilateral T2 hyperintensities of the putamen and caudate nuclei in all affected individuals (white arrowheads) associated with atrophy in all except three affected individuals (F3-P1, F4-P1 and F5-P1). Focal thalamic signal alterations are present in affected individuals F2-P2, F3-P1 and F4-P1 (white dashed arrows). Involvement of the accumbens nuclei is indicated by white empty arrows. Additional symmetric midbrain T2 hyperintensities (black arrowheads) are depicted in affected individuals F1-P2 and F3-P1. Asymmetric foci of restricted diffusion are present at the level of the basal ganglia (white thin arrows) in individuals F1-P2, F2-P2 and F3-P1. Moderate-to-severe white matter volume loss with enlarged ventricles (black asterisks) are associated with multiple areas of supratentorial cortico-subcortical signal alterations and atrophy (white thick arrows) in patients F1-P1, F2-P1 and F5-P1 at first MRI. White matter cystic rarefaction is noted in the deep white matter in proband F1-P1 and in the subcortical regions in F2-P1. F#, family; HC, healthy control; P#, patient; V#, *NDUFA9* variant; WT, wild-type *NDUFA9* allele.

To facilitate functional studies, punch skin biopsies were obtained from proband F1-P1 (Fig. 1A), his affected brother (F1-P2), his unaffected mother (F1-P3), and proband F3-P1, from which dermal fibroblast cultures were established.

The study was conducted in accordance with the principles of the Declaration of Helsinki and received approval from local Ethics Committees. Written informed consent for video acquisition and genetic testing as well as for the publication of clinical, genetic data, and video recordings was obtained from the parents or guardians of the affected individuals.

### Literature review

A systematic literature review was conducted using PubMed, Web of Science, and Google Scholar to identify published cases carrying biallelic *NDUFA9* variants with individual phenotypic information. The search term ‘NDUFA9’ was applied on 15 June 2025. Predetermined phenotypic and genotypic data were extracted from the identified studies and subsequently merged with information from our study cohort for comprehensive analysis.

### Analysis and interpretation of *NDUFA9* variants

Frequencies of newly and previously identified *NDUFA9* variants in the healthy population were retrieved from gnomAD v4.1.0 and other open-source and private databases. Pathogenicity prediction of all *NDUFA9* variants was assessed using multiple tools, including the Combined Annotation-Dependent Depletion (CADD; https://cadd.gs.washington.edu/snv), Deleterious Annotation of genetic variants using Neural Networks (DANN; accessed through Franklin, https://franklin.genoox.com), Polymorphism Phenotyping v2 (PolyPhen-2; http://genetics.bwh.harvard.edu/pph2/), Sorting Intolerant From Tolerant Databases for Genome (SIFT 4G; https://sift.bii.a-star.edu.sg/sift4g/public//Homo_sapiens/); MutationTaster (http://www.mutationtaster.org/), and Rare Exome Variant Ensemble Learner (REVEL; https://sites.google.com/site/revelgenomics/), to predict the impact of variants on the protein structure and function. We examined the conservation of substituted amino acid residues using Genomic Evolutionary Rate Profiling (GERP; accessed through Franklin, https://franklin.genoox.com) and visual multiple sequence alignment of NDUFA9 across different species. Variants were finally classified according to the American College of Medical Genetics and Genomics (ACMG)/Association for Molecular Pathology (AMP) guidelines.^18^

### NDUFA9 protein modelling

The human complex I model (PDB 5XTD)^19^ was used as input for the University of California, San Francisco (UCSF) ChimeraX,^20^ where the *rotamers* or *swapaa* functions were used to introduce NDUFA9 variants, with residues mutated to glycine using the latter. Rotamers of the highest prevalence in the ChimeraX library were chosen, with obvious clashing conformations not considered. The residues within 4 Å of the mutated residue were inspected for interactions. All images were generated in UCSF ChimeraX and organized in Affinity Designer 2.

### Dermal fibroblast culture

Dermal fibroblasts were cultured in medium containing Dulbecco's Modified Eagle Medium (DMEM) supplemented with 1 mM pyruvate and 4.5 g/L glucose (ThermoFisher Scientific), 10% bovine foetal serum (ThermoFisher Scientific), 50 µg/mL uridine (Sigma), 1× non-essential amino acids (ThermoFisher Scientific), 1% penicillin/streptomycin (ThermoFisher Scientific) at 37°C in 5% CO_2_.

### Fibroblast preparation, SDS-PAGE and Western blot analysis

Steady-state levels of important complex I subunits and other OXPHOS components were assessed by sodium dodecyl sulfate-polyacrylamide polyacrylamide gel electrophoresis (SDS-PAGE) of study participants and healthy control fibroblast cell lysates as previously described.^21^ Proteins of interest were bound by overnight incubation at 4°C with antibodies against NDUFA9, NADH:ubiquinone oxidoreductase core subunit V1 (NDUFV1), NADH:ubiquinone oxidoreductase subunit B8 (NDUFB8; all complex I), succinate dehydrogenase complex iron sulphur subunit B (SDHB; complex II), ubiquinol-cytochrome c reductase core protein 2 (UQCRC2; complex III), mitochondrially encoded cytochrome c oxidase II (MT-CO2; complex IV), ATP synthase F1 subunit alpha (ATP5A; complex V) and glyceraldehyde-3-phosphate dehydrogenase (GAPDH) as a loading control, followed by horseradish peroxidase (HRP)-conjugated secondary antibodies (Dako Cytomation) and visualized using enhanced chemiluminescence (ECL)-prime (GE Healthcare) and BioRad ChemiDoc MP with Image Lab software.

### Mitochondrial preparation and blue native electrophoresis

To assess the assembly of respiratory chain complexes, blue native polyacrylamide gel electrophoresis (BN-PAGE) of enriched mitochondrial preparations solubilized with n-Dodecyl β-D-maltoside from patient-derived fibroblasts and age-matched control cell-lines were performed as previously described.^22^

### Statistical analysis

Statistics were performed using Microsoft Excel. For descriptive statistics, results are provided as fractions and/or percentages for dichotomous variables and as mean with range for continuous variables. Results of mitochondrial complex I enzymatic activity are reported as means ± standard deviations (SD).

## Results

We identified eight new cases of *NDUFA9*-related mitochondrial disease belonging to six unrelated families of Asian, European and North African ancestry (Fig. 1A). Videos of seven new affected individuals were available (Supplementary Video 1). Pheno-genotypic features of our cohort and four unrelated cases belonging to four families identified through the systematic literature review are detailed in Table 1 and Supplementary File 1.

**Table 1:** Summary of clinical, neuroradiological and genetic features of new and previously reported cases of *NDUFA9*-related mitochondrial disease

Family	F1		F2		F3	F4	F5	F6	F7	F8	F9	F10
Patient	P1	P2	P1	P2	P1	P1	P1	P1	P1	P1	P1	P1
Gender	M	M	F	F	M	F	M	M	M	M	M	F
Age at onset	18 mo	26 mo	4 yr	8 yr	7 mo	N/A	3 mo	2.5 yr	Neonatal	7 yr	Neonatal	9 yr
Age last assessment	19 yr	13 yr (†)	8 yr	12 yr	8 mo (†)	2.5 yr	33 mo	15 yr	1 mo (†)	Mid-40s	4 mo (†)	26 yr
Ethnicity	Algerian	Algerian	Iranian	Iranian	White British	Iranian	Indian	Afghan	Iraqi	Chinese	Iranian	Indian
Consanguinity	Y	Y	Y	Y	N	Y	Y	Y	Y	N	Y	Y
Family history	Y	Y	Y	Y	N	N	?	N	N	N	N	N
Perinatal issues	Y	Y	N	N	Y	N	N	N	Y	N	Y	N
NDD	Y	Y	Y	Y	Y	Y	Y	Y	N/A	N	Y	N
Clinical manifestations												
*Regression*	Y	Y	Y	N	Y	Y	N/A	Y	N/A	N	N	N
*ID*	Y (mod)	Y (mod)	Y (sev)	Y (mod)	N/A	Y (sev)	Y (sev)	N/A	N/A	N	N/A	N
*Dystonia*	Y	Y	Y	Y	Y	Y	Y	Y	Y	Y	N	Y
*Feeding issues/Dysphagia/FTT*	Y	Y	Y	Y	N/A	Y	Y	Y	N/A	Y	Y	N
*Pyramidal signs*	Y	Y	Y	Y	Y	Y	Y	Y	N/A	N	Y	N
*Dysarthria/Anarthria*	Y	Y	Y	Y	N/A	N/A	N/A	N	N/A	Y	N/A	N
*Muscle weakness/atrophy*	Y	Y	Y	Y	N	Y	Y	Y	N	N	Y	N
*Seizure(s)*	Y	Y	Y	Y	Y	N	Y	Y	N	N	N	N
Abnormal brain MRI	Y	Y	Y	Y	Y	Y	Y	Y	Y	Y	Y	Y
Increased lactate	Y (bl)	Y (bl)	Y (bl)	N/A	Y (bl, CSF)	N/A	Y (bl)	N/A	Y (bl, ur)	Y (bl)	Y (bl, CSF)	N
Variant(s) *NDUFA9* (ENST00000266544.10; NM_005002.5)	Hom c.1061G > A p.(R354Q)	Hom c.1061G > A p.(R354Q)	Hom c.1061G > A p.(R354Q)	Hom c.1061G > A p.(R354Q)	C-Het c.1078C > G p.(R360G) & c.800G > T p.(G267V)	Hom c.1061G > A p.(R354Q)	Hom c.710A > T p.(K237I)	Hom c.1079G > T p.(R360L)	Hom c.962G > C p.(R321P)	Hom c.1078C > T p.(R360C)	Hom c.1069C > G p.(R357G)	Hom c.727G > A p.(V243I)
Reference(s)	This article	This article	This article	This article	This article	This article	This article	This article	^14,15^	^15^	^16^	^17^

See also Fig. 1A and Supplementary File 1.

bl, blood; C-Het, compound heterozygote; F, female; F#, family; FTT, failure to thrive; Het, heterozygote; Hom, homozygote; ID, intellectual disability; M, male; mo, month(s); mod, moderate; N, no (absent); N/A, not applicable/not available; NDD, neurodevelopmental delay; P#, patient; sev, severe; ur, urine; Y, yes (present); yr, year(s); †, age at death.

### Phenotypic features

Among eight new and four previously published *NDUFA9* cases, 8/12 (66.7%) were males and 4/12 (33.3%) were females. Parental consanguinity was reported in all but two affected individuals (10/12, 83.3%; F3-P1, F8-P1). Two of 10 (20%) families had more than one similarly affected member (F1 and F2). Three probands (F3-P1, F7-P1 and F9-P1) died during infancy (range: 1–8 months), and one other affected individual (F1-P2) died at the age of 13 years. In 8/12 (66.7%) affected individuals who were alive at the time of the study or reporting, the age at last assessment ranged from 2.5 years to mid-40s.

Three of 12 (25.0%) individuals were born preterm. Delivery via caesarean section was reported in 1/12 (8.3%) case. Perinatal issues were described in 5/12 (41.7%) affected individuals, including respiratory distress/insufficiency (three cases), hypotonia (two cases) and bradycardia (one case). Neurodevelopmental delay followed by motor deterioration and seizures were the most common presenting features. Among 10/12 (83.3%) cases who survived beyond the age of 4 months, motor and/or speech and language developmental delay was observed in nine (90.0%). Among 9/12 (75.0%) cases who survived beyond infancy, five (55.5%) individuals never attained unsupported sitting, and six (66.7%) cases were reported with moderate-to-severe intellectual disability. Onset of neurological deterioration invariably occurred within the first decade of life (range: neonatal period—9 years) and prior to age the age of 4 years in 9/12 (75.0%) cases. When neurological deterioration began, worsening of motor performance or motor regression was reported as the earliest manifestation in 8/12 (66.7%) cases and seizures in 3/12 (25.0%).

Among 10/12 (83.3%) cases surviving beyond the age of 4 months, main clinical manifestations encompassed dystonia (10/10, 100%), feeding difficulties/dysphagia/failure to thrive and pyramidal tract signs (8/10, 80%), seizures and muscle weakness/atrophy (7/10, 70%), and dysarthria/anarthria/stuttering (6/10, 60%). Dystonia was generalized since onset in 7/10 cases (70%), whereas it started in the distal lower limbs and progressed to a generalized form in 2/10 cases (20%) and remained multifocal in 1/10 case (10%). Of seven individuals with seizures, five (71.4%) experienced only generalized seizures, and two (28.6%) had both focal motor and generalized seizures. Seizures were of tonic type in most cases, and status epilepticus was reported in one case 1/7 case (14.3%) during a respiratory infection (Table 1 and Supplementary File 1).

Minor phenotypic features include strabismus (3/10, 30%), scoliosis (2/10, 20%), ataxia (2/10, 20%), and peripheral sensorimotor neuropathy (1/10, 10%). In three cases who died during infancy, one had retinopathy of prematurity, one had retinitis pigmentosa, and one had hearing loss. Additional clinical information is detailed in Table 1 and Supplementary File 1.

Among cases with biochemical tests available, blood lactate was increased in 8/9 (88.9%) where available, and CSF lactate was raised in 2/5 (40.0%) where available. Five affected individuals underwent respiratory chain enzyme testing in dermal fibroblasts and/or muscle tissue, with confirmation of isolated complex I deficiency in all and detection of a complex I stalled assembly intermediate in one individual (F3-P1).

Brain MRI examinations were available for review in all eight new affected individuals. MRI scans were performed at a mean age of 3.1 years (range: 5 months–7 years). All individuals showed bilateral symmetric or asymmetric signal alterations of the basal ganglia (8/8, 100%) with associated mild-to-severe atrophy in four cases (50.0%) and swelling in two individuals (25.0%; Fig. 1B). In particular, the putamen and caudate nuclei were involved in 8/8 cases (100%), the accumbens nuclei in 5/8 (62.5%), the thalami in 3/8 (37.5%) and the globi pallidi in 3/8 individuals (37.5%). Diffusion weighted images (DWI) were available in three cases, showing foci of restricted diffusion in all individuals at the level of the putamina (*n* = 2; 66.7%), caudate nuclei (*n* = 1; 33.3%), accumbens nuclei (*n* = 1; 33.3%), and thalamus (*n* = 1; 33.3%). In 4/8 individuals (50.0%), there was moderate-to-severe white matter volume loss and signal alterations, with thin corpus callosum and enlarged ventricles; in three of these individuals, there were also multiple regions of cortical-subcortical signal alterations and atrophy associated with white matter cavitations (*n* = 2 in the deep white matter and *n* = 1 in the subcortical white matter); these findings were consistent with a leukodystrophy with cystic rarefaction associated with multifocal cortical atrophy. Finally, in 3/8 individuals, bilateral symmetric signal alterations were noted in the brainstem, including the central tegmental tracts and cerebral peduncles (*n* = 3), periaqueductal gray matter (*n* = 2), and posterior medulla (*n* = 1). In two individuals (F1-P1 and F1-P2), follow-up brain MRI performed at the age of 17 and 12 years, respectively showed marked progression of the cerebral and basal ganglia atrophy associated with significant worsening of the leukodystrophy (Supplementary File 2). Brain MR spectroscopy did not show lactate or other metabolite peak in two cases where available.

EEG showed multifocal epileptic activity or was normal at different stages in individuals with history of seizures (Supplementary File 1). Ophthalmological assessment was performed in two affected individuals who survived into the second decade of life; one individual showed bilateral papillary pallor, while the other had normal findings. Visual evoked potentials were normal in two probands where performed. EMG/NCS was performed in three affected individuals, showing axonal sensorimotor neuropathy in one and being normal in two.

### Genetic findings

**Figure 2:**
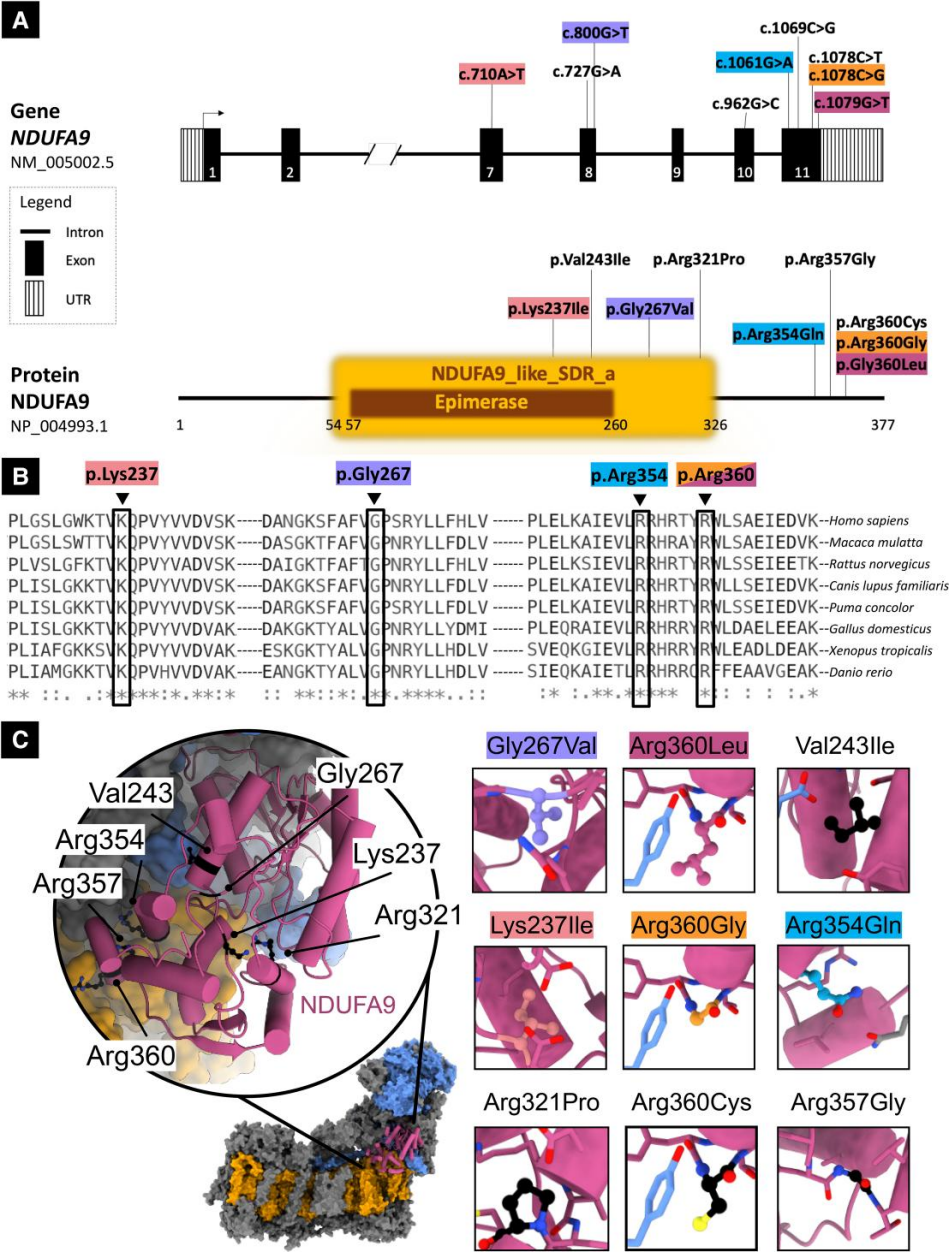
**Location, computational analysis and conservation of *NDUFA9* variants.** (**A**) Schematic of *NDUFA9* and its protein product with five new variants identified in this study and four variants identified in previously reported families. *Upper part*. Schematic of *NDUFA9* with variants identified in this study (coloured; see also Fig. 1A and Supplementary File 3) and previously reported. Introns are not to scale. Exon numbers are according to the canonical transcript (NM_006814.5). *Lower part*. Schematic of NDUFA9 protein with amino acid changes linked to disease (NP_006805.2). (**B**) Interspecies alignment showing strong evolutionary conservation of the amino acids affected by the new *NDUFA9* missense variants identified in this study across species, down to invertebrates. (**C**) *In silico* analysis of human complex I NDUFA9 variants. NDUFA9 (pink) is one of 31 supernumerary subunits (others grey) of mitochondrial complex I that wrap around the core subunits, which compose a membrane arm (orange) and a hydrophilic, matrix arm (blue). The human complex I model (PDB 5XTD) shows how NDUFA9 helps to bind the hydrophilic domain to the membrane through protein–lipid interactions. Inset of NDUFA9 demonstrates the wild-type amino acid locations and conformations (black ball and sticks). Specific mutations of these residues are indicated and a prediction of their rotamer conformation shown (ball and stick), with immediate vicinity residues (within 4 Å) also displayed to highlight disruption to electrostatic interactions (Lys237Ile, Arg321Pro, Arg354Gln, Arg357Gly), lipid binding (Arg360Leu, Arg360Gly, Arg360Cys), as well as increased clashing (Gly267Val, Val243Ile). SDR, short-chain dehydrogenase/reductase; UTR, untranslated region.

We identified five new missense variants in *NDUFA9* (ENST00000266544.10; NM_005002.5; NP_006805.2; Fig. 2A), thereby expanding the known genotypic spectrum of *NDUFA9*-related mitochondrial disorder. Previously, only four missense variants had been reported [c.962G > C p.(Arg321Pro), c.1078C > T p.(Arg360Cys), c.1069C > G p.(Arg357Gly), c.727G > A p.(Val243Ile)]. Among the newly identified *NDUFA9* variants, one variant recurred in three unrelated pedigrees [c.1061G > A p.(Arg354Gln), F1-F2-F4], and two variants were detected in two unrelated pedigrees [c.1078C > G p.(Arg360Gly), F3; c.1079G > T p.(Arg360Leu), F6] and affected the same nucleotide of another previously reported variant [c.1078C > T p.(Arg360Cys), F8], which therefore emerges as a possible mutational hotspot (Fig. 2A).^15^ In all new cases with genomic DNA available, segregation analysis revealed that biallelic *NDUFA9* variants co-segregated with neurological disease phenotypes, whereas unaffected parents and siblings of affected individuals harboured one heterozygous *NDUFA9* variant (Fig. 1A). Among eight new affected individuals, seven were homozygotes and one compound heterozygote for *NDUFA9* variants (Fig. 1A). Variants analysis is detailed in Supplementary File 3. All *NDUFA9* variants identified in this study were not observed in the homozygous state or ultra-rare in the heterozygous state in gnomAD v4.1.0, other open-source population databases and private next-generation sequencing repositories (Supplementary File 3). NDUFA9 variants identified in this study showed strong evolutionarily conservation across species down to invertebrates (Fig. 2B). All *NDUFA9* variants were predicted with a damaging effect according to most *in silico* prediction tools (Supplementary File 3). All *NDUFA9* variants had a high CADD Phred score (range: 23.6–33) and REVEL score ranging from 0.27 to 0.94. Pathogenicity prediction tools such as PolyPhen-2, SIFT 4G and MutationTaster predicted the functional impact of all *NDUFA9* missense variants to be damaging/deleterious in most cases (Supplementary File 3). According to the ACMG/AMP guidelines, *NDUFA9* variants identified in new cases were classified as pathogenic [c.800G > T p.(Gly267Val)], likely pathogenic [c.1061G > A p.(Arg354Gln); c.1078C > G p.(Arg360Gly) and variant of uncertain significance [c.710A > T p.(Lys237Ile); c.1079G > T p.(Arg360Leu)]. No additional biallelic variants of (likely) pathogenic significance were identified in the affected individuals.

### Potential effects of the variants to NDUFA9 folding and binding

NDUFA9 variants can introduce disruption to electrostatic and packing interactions, which can lead to protein misfolding and/or disruption to binding interfaces. Among nine new and previously reported variants in NDUFA9, six replace Arginine with Leucine, Glycine, Glutamine, Cysteine or Proline residues, and one is a Lysine to Isoleucine. All substitutions remove the positive charge and affect stability of negative amino acids in structurally important ion pairs (Fig. 2C, Lys237Ile and Arg321Pro). Additionally, Arginine has important interactions with negatively charged phospholipid headgroups when located on membrane-binding surface, such as Arg360, where they help to embed the subunit and anchor the hydrophilic domain of complex I. Arg360Leu, Arg360Gly and Arg360Cys will all disrupt this capacity. The two variants that do not affect the electrostatics, Gly267Val and Val243Ile, both have detrimental impact by introducing unfavourable clashing interactions due to their larger size, particularly Gly267Val.

### Western blot and biochemical analysis of fibroblasts

**Figure 3:**
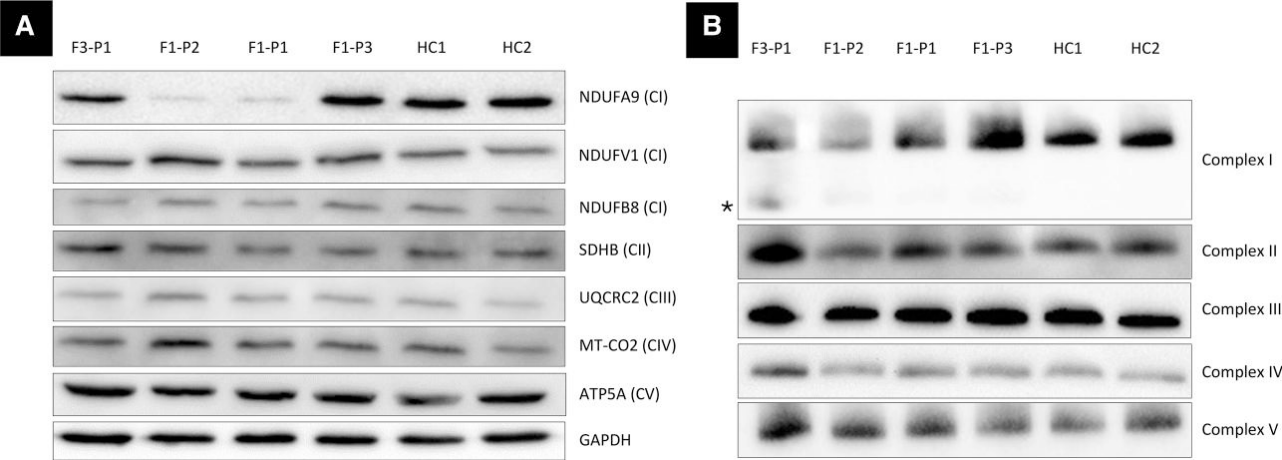
**Western blot and biochemical assessment of lysates from dermal fibroblasts of individuals with biallelic *NDUFA9* variants.** (**A**) Whole-cell (fibroblast) lysates from individuals F3-P1 (affected), F1-P2 (affected), F1-P1 (affected), F1-P3 (unaffected carrier; Fig. 1A) and age-matched healthy controls (HC1, HC2) were analysed by SDS-PAGE. Immunoblotting was performed with antibodies against complex I subunits and several OXPHOS subunits as indicated, with GAPDH (cytosolic protein) utilized as a loading control. C# indicates mitochondrial complexes I to V. (**B**) Mitochondria isolated from cultured dermal fibroblasts from individuals F3-P1 (affected), F1-P2 (affected), F1-P1 (affected), F1-P3 (unaffected carrier; Fig. 1A) and age-matched healthy controls (HC1, HC2) were solubilized in n-dodecyl β-D-maltoside (DDM) and subjected to BN-PAGE and immunoblotting analysis using antibodies directed to various OXPHOS complexes, NDUFB8 (CI), SDHA (CII), UQCRC2 (CIII), MT-CO2 (CIV), ATP5A (CV). The blot probed with an antibody raised against NDUFB8 (CI) revealed the presence of an additional partially assembled complex I intermediate in proband F3-P1 (asterisk). ATP5A, ATP synthase F1 subunit alpha; BN-PAGE, blue native polyacrylamide gel electrophoresis; CI, complex I; CII, complex II; CIII, complex III; CIV, complex IV; CV, complex V; F#, family; GAPDH, glyceraldehyde-3-phosphate dehydrogenase; HC#, healthy control; MT-CO2, mitochondrially encoded cytochrome c oxidase II; NDUFA9, NADH:ubiquinone oxidoreductase subunit A9; NDUFV1, NADH:ubiquinone oxidoreductase core subunit V1; NDUFB8, NADH:ubiquinone oxidoreductase subunit B8; SDHB, succinate dehydrogenase complex iron sulphur subunit B; P#, patient; UQCRC2, ubiquinol-cytochrome c reductase core protein 2. Full-size and uncropped blots/gels are presented in Supplementary File 4.

To further investigate the effect of three *NDUFA9* variants identified in this study on mitochondrial function, we established dermal fibroblast cultures from skin biopsies obtained from two probands (F1-P1, F3-P1), one affected brother (F1-P2) of proband F1-P1 and their asymptomatic mother (F1-P3). SDS-PAGE immunoblotting using antibodies against various structural subunits of complex I revealed an overall reduction in the steady-state levels of NDUFA9 protein in the cells from affected individuals F1-P1 and F1-P2 compared to healthy controls (Fig. 3A; Supplementary File 4), whilst normal levels were detected in cells from F3-P1 and F1-P3. Levels of key subunits of complex II, complex III, complex IV and complex V were all normal (Fig. 3A). Immunoblotting using antibodies conjugated against structural subunits from all OXPHOS complexes showed a loss of fully assembled complex I in cells from F3-P1, F1-P1 and F1-P2, while all other OXPHOS complexes were unaffected (Fig. 3B; Supplementary File 4). Furthermore, analysis of F3-P1 cells appeared to show the presence of a partially assembled, smaller complex I intermediate (Fig. 3B; Supplementary File 4). Cells from F1-P3 revealed a profile indistinguishable from healthy controls. Assessment of mitochondrial respiratory chain enzyme activities revealed isolated complex I deficiency in the fibroblasts from F1-P1 (complex I activity: 0.133 nmols NADH oxidized.min^−1^.unit citrate synthase^−1^) and F3-P1 (0.114 nmols NADH oxidized.min^−1^.unit citrate synthase^−1^), although surprisingly fibroblasts from F1-P2 showed normal complex I activity (0.197 nmols NADH oxidized.min^−1^.unit citrate synthase^−1^; controls 0.197 ± 0.034 [mean ± SD, *n* = 8]); all other respiratory chain complex activities were normal. As expected, no mitochondrial biochemical abnormalities were detected in the fibroblasts from F1-P3 (complex I activity: 0.244 nmols NADH oxidized.min^−1^.unit citrate synthase^−1^).

## Discussion

Isolated complex I deficiency is the most frequent childhood-onset OXPHOS disorder.^11-13^ Most cases present with neurodevelopmental delay and progressive neurological deterioration with clinical manifestations of basal ganglia and/or brainstem dysfunction, elevated blood or CSF lactate, and MRI or pathological evidence of basal ganglia and/or brainstem abnormalities, which leads to the diagnosis of LS.^12^ Presentations not fulfilling all the above-mentioned criteria are labelled as Leigh-like syndrome (LLS). Taken together, disease-causing variants in genes encoding complex I subunits represent the major cause of LS/LLS. However, information on the pheno-genotypic spectrum linked to defects in most individual complex I subunits is scarce.

With the present study, 12 *NDUFA9* cases from 10 families have been reported. *NDUFA9*-related mitochondrial disease shows wide phenotypic variability, with clinical presentation ranging from typical LS with lethality during the neonatal period to childhood-onset, isolated, generalized dystonia with survival into adulthood.^14-17^ Notably, neurological deterioration always started within the first decade of life and was triggered by infections/febrile episodes in few cases. Most *NDUFA9* patients showed neurodevelopmental delay/intellectual disability and complex movement abnormalities, in keeping with the phenotypic spectrum reported for other mitochondrial disorders caused by mutations in nuclear and mitochondrial DNA genes encoding complex I subunits.^23^ In *NDUFA9*-related mitochondrial disease, dystonia was the most prominent motor features, followed by pyramidal tract signs.^23,24^ As recently reviewed by members of our team,^23^ prominent dystonia, most often in the context of a neurodevelopmental disorder, is a common presentation in cases of *NDUFS6*-, *NDUFAF5*-, *NDUFS7*-, *NDUFA12*-,^24^  *NDUFS1*-, *NDUFS4*-, MT-*ND1*-, and MT-*ND3*-related mitochondrial disease. Over two thirds of *NDUFA9* cases had a history of seizures, which had not been reported in previously published *NDUFA9* cases and was however not infrequent in cases of *NDUFS4*-, *NDUFS8*-, *NDUFV1*-, *NDUFAF5*-, *NUBPL*-, MT-*ND1*-, MT-*ND3*-, MT-*ND5*-, and MT-*ND6*-related mitochondrial disease.^23^ Increased blood lactate levels were the most common albeit inconstant biochemical finding. When available, brain MRI always revealed a LS pattern characterized by bilateral, mostly symmetric basal ganglia signal alterations associated with atrophy or swelling and additional foci of restricted diffusion. Similar neuroradiological findings were previously described in three individuals harbouring biallelic *NDUFA9* variants,^14,15,17^ which suggests a selective vulnerability of these anatomical structures. In addition, in two individuals we found other typical neuroimaging manifestations of LS, with bilateral and symmetric periaqueductal gray matter, cerebral peduncles, central tegmental tracts and posterior medulla signal alterations.^25^ Four patients had white matter signal alterations and volume loss, in keeping with a leukodystrophy with additional cystic rarefaction in three cases.^26^ Of note, in two subjects, the cysts were large and located in the deep white matter, as previously described in other mitochondrial disorders.^26^ Conversely, in one case, the cystic rarefaction was subtle and located in the subcortical regions, an atypical finding in mitochondrial leukodystrophies.^26^ Interestingly, the previously unreported association of a cavitating leukodystrophy with early and prominent cortical atrophy expands the neuroimaging phenotype of *NDUFA9*-related disease.

From a genotypic perspective, all nine *NDUFA9* variants linked to human disease so far are missense mutations, which might suggest that complete loss of function is not compatible with complex I assembly and/or functioning and ultimately with survival in humans. Intriguingly, none of the NDUFA9 variants hitherto reported maps at the N-terminal of the protein, where the presequence addressing the synthetized protein to mitochondria is located. On the contrary, most variants identified in human *NDUFA9* cases lie in the last exon, including three variants affecting the residue Arg360, which we herein highlight as a possible mutational hotspot.

NDUFA9 sits at the interface of the membrane and matrix arms of mitochondrial complex I and plays a role in securing the hydrophilic domain to the membrane.^6^ This is achieved by the binding of negative phospholipids to largely positively charged amino acids (e.g. Lysine and Arginine) found on NDUFA9 membrane-interacting face. In this study, protein modelling revealed that NDUFA9 missense variants hitherto reported are implicated in protein misfolding through clashing or unfavourable electrostatic interactions. Notably, six NDUFA9 variants affect wild-type Arginine residues. Arginine has an important role in anchoring the NDUFA9 subunit and stabilizing the enzyme through binding and remodelling the phospholipid bilayer. NDUFA9 amino acid changes affecting these residues could interfere with the recruitment of the membrane-bound substrate ubiquinone-10 and ultimately disrupt complex I function.^7,8^

Our experiments investigating mitochondrial function in fibroblasts from three new *NDUFA9* cases invariably showed loss of fully assembled complex I. Furthermore, decreased steady-state levels of NDUFA9 protein were detected in two related homozygotes for the variant Arg354Gln. On the contrary, levels observed in one compound heterozygote for the variants c.1078C > G p.(Arg360Gly) and c.800G > T p.(Gly267Val) were similar to healthy controls. Intriguingly, the same affected individual showed a partially assembled, smaller complex I intermediate. This observation parallels *in vitro* evidence that NDUFA9 contributes to stability of a later stage complex I assembly in *NDUFA9* knockout in HEK293T cells from a previous study.^6^

In conclusion, our study provides further evidence of the pheno-genotypic spectrum associated with biallelic *NDUFA9* variants and mitochondrial complex I deficiency. This evidence supports the inclusion of *NDUFA9* variants in the diagnostic workup of complex movement disorders encompassing dystonia and/or pyramidal features and a LS pattern of brain MRI signal abnormalities. Future case reporting may expand our understanding of *NDUFA9*-related mitochondrial disease and help establish whether genotype-phenotype correlations exist. To date, such correlations have been hampered by the limited number of reported cases and variants, most of which are private.

## Supplementary Material

fcaf369_Supplementary_Data

## Data Availability

The authors confirm that the data supporting the findings of this study are available within the article, its Supplementary material and from the corresponding authors, upon reasonable request. No code was generated or used for this study.
