# Supplementary material for: Biallelic NDUFA9 variants cause a progressive neurodevelopmental disorder with prominent dystonia and mitochondrial complex I deficiency
Source: Brain Commun. 2025 Sep 23;7(5):fcaf369. doi: 10.1093/braincomms/fcaf369 (PMC12507085; doi:10.1093/braincomms/fcaf369)
Supplement: fcaf369_Supplementary_Data [file fcaf369_supplementary_data.zip › Supplementary_Video_1_Legend.pdf]

Supplementary Video 1 Legend

Segment 1. A 19-year-old Algerian male (F1-P1) presented with generalised mobile dystonia. Dystonia mainly affected his neck, trunk, upper limbs and feet. There was strabismus, intermittent facial grimace and episodic head drop. He could only stand on his tiptoes with bilateral support. There was diffuse hypotrophy of limb muscles.
Segment 2. A 13-year-old Algerian male (F1-P2) showed generalised mobile dystonia with prominent head and trunk extension. There was strabismus and intermittent facial grimace. He had also intermittent head drop and diffuse muscle hypotrophy.
Segment 3. An 8-year-old Iranian female (F2-P1) manifested with generalised dystonia with prominent involvement of the left hemibody, poor head and trunk control and behavioural issues. Tendon reflexes were normal. Plantar response was downgoing bilaterally.
Segment 4. A 12-year-old Iranian female (F2-P2) showed generalised dystonia with main involvement of her hands and feet. There were no pyramidal tract signs.
Segment 5. A 2.5-year-old Iranian female (F4-P1) presented with gait impairment and recurrent falls due to generalised dystonia prominently involving her right hemibody, facial grimace and jaw-opening dystonia, mixed dystonia and spasticity in her right limbs with right hand clenching and right foot posturing.
Segment 6. A 3-year-old Indian female (F5-P1) showed dystonia with prominent involvement of her lower limbs. She could not sit still due to extensor neck and truncal muscle weakness. There was repetitive head drop.
Segment 7. A 15-year-old Afghani male (F6-P1) presented with short stature, generalised muscle hypotrophy, bilateral ptosis, myopia, strabismus, intermittent dystonic facial grimace, dystonic posturing of fingers and right hand and generalised dystonia with prominent involvement of his right limbs. He showed furniture and toe walking with increased muscle tone in his lower limbs and foot intoeing.
