# Supplementary material for: Biallelic NDUFA9 variants cause a progressive neurodevelopmental disorder with prominent dystonia and mitochondrial complex I deficiency
Source: Brain Commun. 2025 Sep 23;7(5):fcaf369. doi: 10.1093/braincomms/fcaf369 (PMC12507085; doi:10.1093/braincomms/fcaf369)
Supplement: fcaf369_Supplementary_Data [file fcaf369_supplementary_data.zip › Supplementary_File_2.pdf]

Supplementary File 2. Follow-up neuroimaging features in *NDUFA9*-related mitochondrial disease.

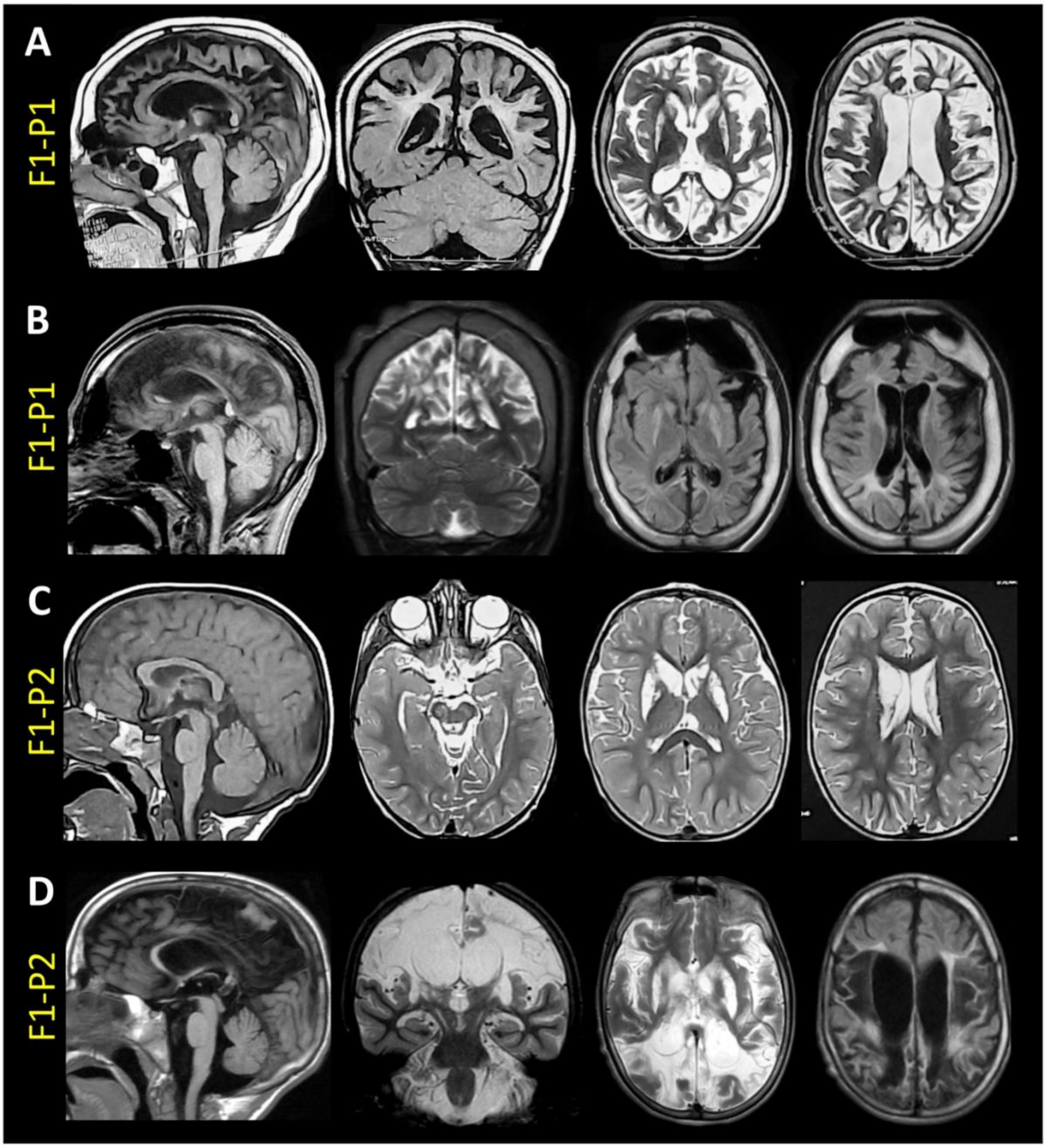

Brain MRI examinations of proband F1-P1 performed at 7 years **[A]** and 17 years of age **[B]** and affected individual F1-P2 at 4 years **[C]** and 12 years of age **[D]**. In both cases, follow-up MRI depicted marked progression of cerebral atrophy with large areas of white matter cystic rarefaction, severe white matter volume loss with ventricular enlargement, cortical atrophy and basal ganglia atrophy. Legend: F# = family; P# = patient (see Figure 1A).
