# Supplementary material for: Biallelic NDUFA9 variants cause a progressive neurodevelopmental disorder with prominent dystonia and mitochondrial complex I deficiency
Source: Brain Commun. 2025 Sep 23;7(5):fcaf369. doi: 10.1093/braincomms/fcaf369 (PMC12507085; doi:10.1093/braincomms/fcaf369)
Supplement: fcaf369_Supplementary_Data [file fcaf369_supplementary_data.zip › Supplementary_File_4.pdf]

Supplementary File 4. Full-size and uncropped blots/gels presented in Figure 3A-B.

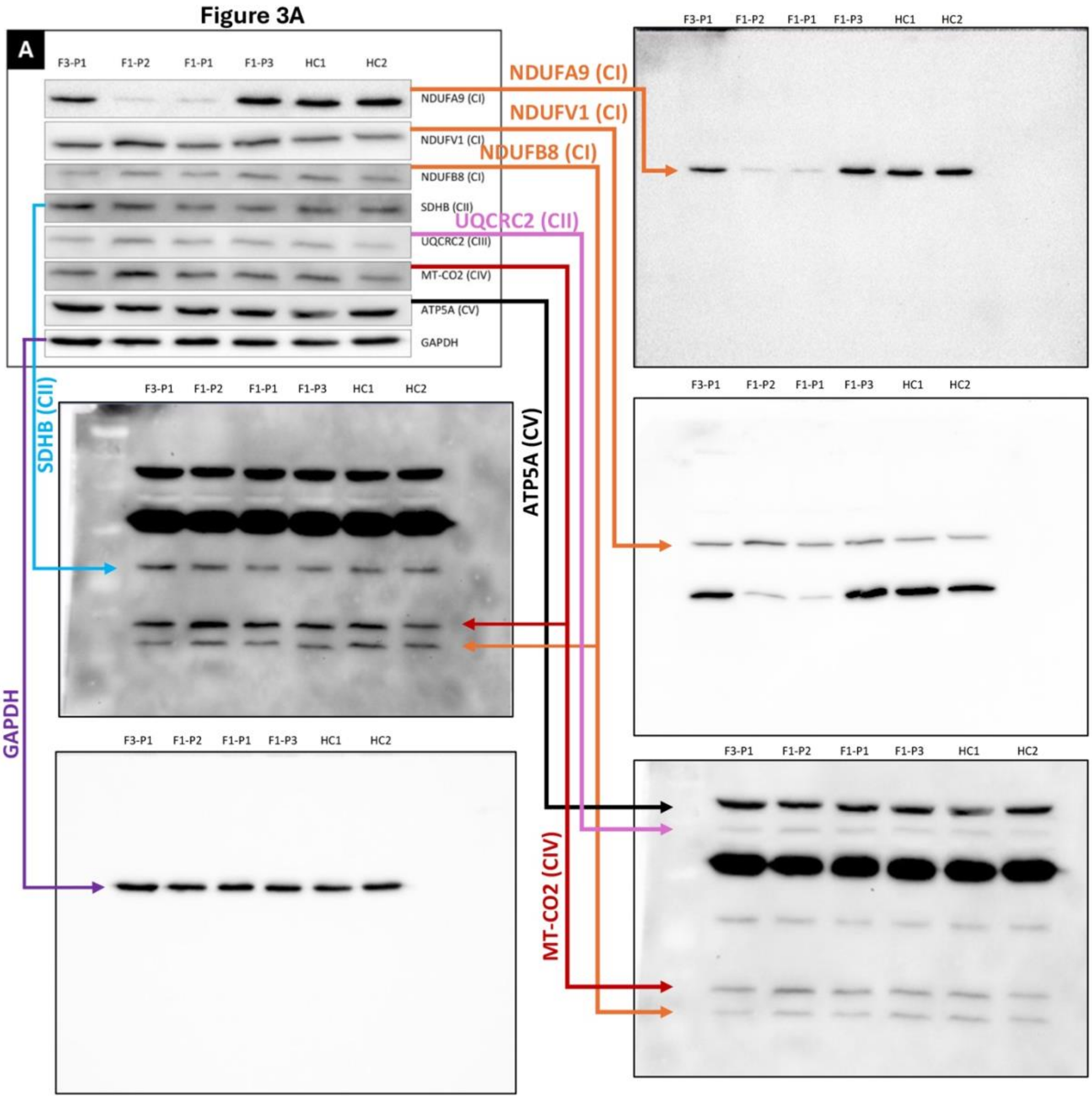

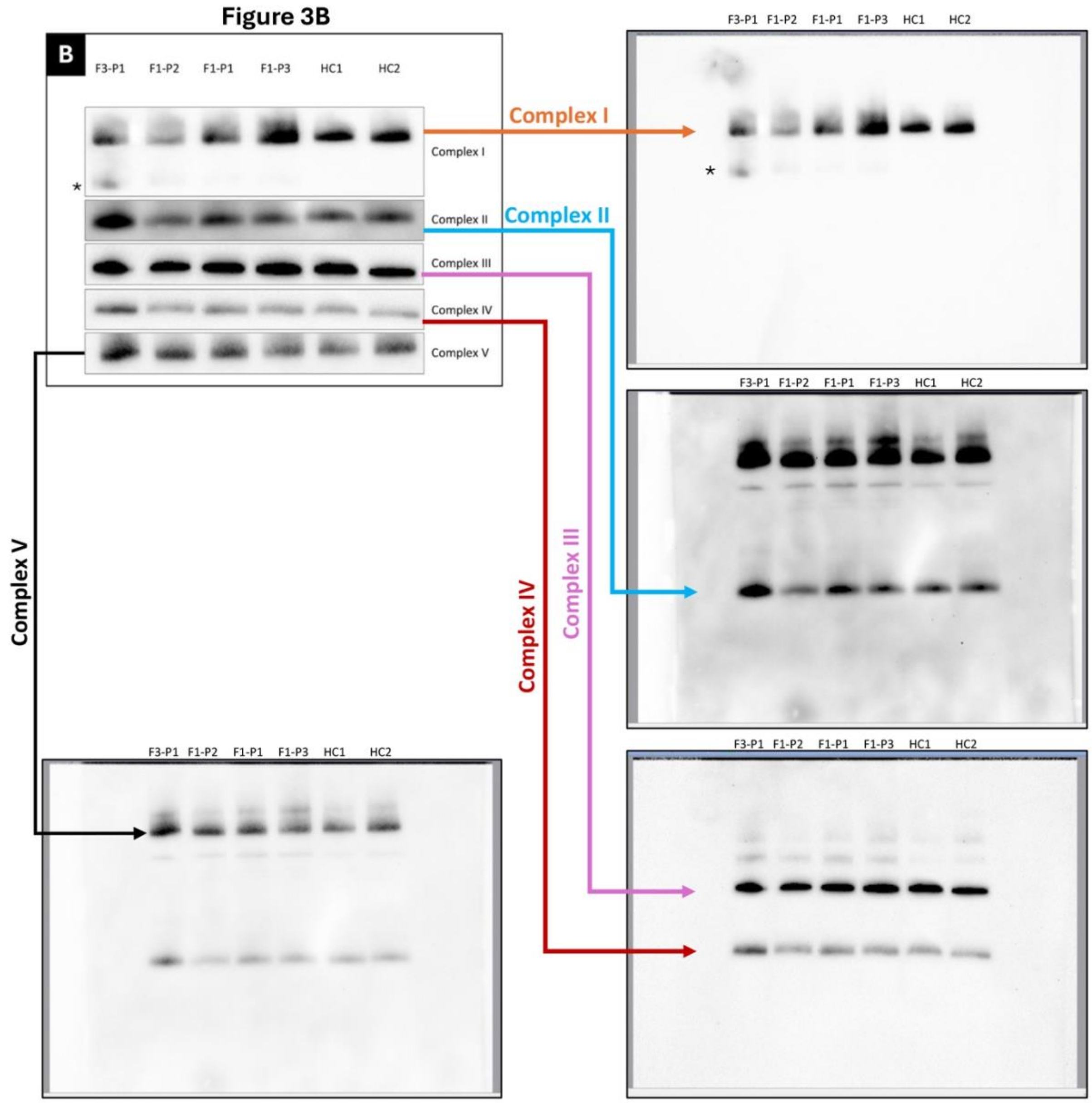

Legend: ATP5A = ATP synthase F1 subunit alpha; CI = complex I; CII = complex II; CIII = complex III; CIV = complex IV; CV = complex V; F# = family; GAPDH = glyceraldehyde-3-phosphate dehydrogenase; HC# = healthy control; MT-CO2 = mitochondrially encoded cytochrome c oxidase II; NDUFA9 = NADH:ubiquinone oxidoreductase subunit A9; NDUFV1 = NADH:ubiquinone oxidoreductase core subunit V1; NDUFB8 = NADH:ubiquinone oxidoreductase subunit B8; SDHB = succinate dehydrogenase complex iron sulfur subunit B; P# = patient; UQCRC2 = ubiquinol-cytochrome c reductase core protein 2.
